# Recent Evidence on the Impact of Ramadan Diurnal Intermittent Fasting, Mealtime, and Circadian Rhythm on Cardiometabolic Risk: A Review

**DOI:** 10.3389/fnut.2020.00028

**Published:** 2020-03-11

**Authors:** Ahmed S. BaHammam, Aljohara S. Almeneessier

**Affiliations:** ^1^Department of Medicine, The University Sleep Disorders Center, College of Medicine, King Saud University, Riyadh, Saudi Arabia; ^2^Department of Family Medicine, College of Medicine, King Saud University, Riyadh, Saudi Arabia

**Keywords:** intermittent fasting, sleep, circadian rhythms, cardiometabolic disease, Ramadan, oxidative stress, proinflammatory markers

## Abstract

In this article, we reviewed recent data that examined the relationship of circadian rhythm, mealtime, and intermittent fasting with the risk of cardiometabolic dysfunction. We also examined the effect of their interactions on cardiometabolic risks. Furthermore, since major differences exists between Ramadan diurnal intermittent fasting compared to other forms of experimental intermittent fasting, in this article, we further restricted the discussion to Ramadan diurnal intermittent fasting. PubMed and Google Scholar databases were searched using “intermittent fasting,” “time-restricted feeding,” “fasting,” “mealtime,” “circadian rhythm,” and “cardiometabolic risk,” focusing on human studies published after 2013. Recent evidence indicates that meal timing may influence circadian rhythm, as a result, it may also directly or indirectly impact cardiometabolic risk. In humans, several studies suggested that late mealtime is related to an increased risk of poor cardiometabolic health. Nevertheless, large clinical interventional studies are required to assess causality between late mealtime and cardiometabolic morbidity. Currently, evidence indicates that Ramadan diurnal intermittent fasting has several beneficial effects that may reduce the risk of cardiometabolic disorders, such as weight reduction, improvement in lipid profile and glycemic control, reduction in proinflammatory markers, and oxidative stress. Nevertheless, several changes in daily lifestyle routine, happening during the Ramadan month, may affect the all measured markers of cardiometabolic diseases. Summarily, no definitive conclusion about the impact of Ramadan intermittent fasting on oxidative stress can be formulated. Therefore, large, well-designed studies, which control for various confounding factors are required to assess the influence of Ramadan diurnal intermittent fasting on markers of cardiometabolic risk and disorders.

## Introduction

Cardiometabolic disorders, a major health problem, is among the main causes of increased morbidity and mortality (1). Cardiometabolic risk denotes risk factors that increase the chances of developing vascular events or developing diabetes and obesity. This conception comprises diseases, environmental, behavioral and genetic factors, such as hypertension, dyslipidemia, poor eating habits, and smoking, in addition to recently described risk factors, such as central obesity, systemic inflammation, and genetics (2, 3).

Important under-recognized factors that have recently been linked to cardiometabolic risk include; intermittent fasting (IF), time-restricted feeding (TRF), mealtime, and circadian rhythm (4). Disturbances of circadian rhythm disrupt body functions and may increase the risk of cardiometabolic dysfunction (5). On the other hand, changes in the mealtime, such as increasing caloric intake at night, have recently been linked to cardiometabolic health in humans (6, 7). In addition, recent data indicate that IF may have positive effects on cardiometabolic function and may reduce the risk of cardiometabolic diseases. In this article, we critically reviewed studies that examined the effect of IF, mealtime, and circadian rhythm; in addition to their interactions on cardiometabolic risks.

## Search Methods

PubMed and Google Scholar databases were searched using the following words “intermittent fasting,” “time-restricted feeding,” “fasting,” “mealtime,” “circadian rhythm,” and “cardiometabolic risk.” We focused on human studies; however, important and relevant animal studies were reported in this review. In this review, we mainly discussed studies published after 2013; though, relevant papers published before that date were included.

## Circadian Clock and Cardiometabolic Risk

All body organs, tissues and cells are under the control of a biologic clock and follow a circadian pattern. Based on the anatomical location, biologic clocks can be separated into peripheral and central clocks.

The central biologic clock is in the hypothalamus and is known as the suprachiasmatic nucleus (SCN). However, each cell in the body has peripheral clocks (8). Biologic clocks maintain the normal tissue functioning by regulating the expression of tissue-specific genes (8).

The biologic clock of the body needs to be entrained daily to achieve external synchrony between the body functions and the external natural time, and internal synchrony between the central and peripheral body clocks (8). The SCN is entrained mainly by light, while peripheral clocks are influenced by neurohormonal factors and mealtime (7, 9).

Studies have shown that desynchrony of the circadian system predisposes to several cardiometabolic dysfunctions, such as glucose tolerance impairment, reduced sensitivity to insulin, elevated proinflammatory cytokines, increased arterial blood pressure, and reduced energy expenditure, which results in obesity (5). The effect of circadian misalignment on cardiometabolic risk is supported by epidemiologic data linking shift work with a higher risk of developing cardiometabolic disorders, such as obesity, diabetes, and cardiovascular diseases (10–12).

Recently, several investigators explored the relationship of acute disruption of circadian rhythm and mealtime with cardiometabolic risk in healthy volunteers. In a well-designed study that aimed to assess the acute impact of misalignment between mealtime, sleep/wake pattern, and internal circadian rhythm on some markers of cardiometabolic health, 10 adults were subjected to a 10-day forced desynchrony protocol where the participants ate iso-caloric meals and slept during a recurrent 28-h day cycle (13). This protocol required feeding and sleeping for about 12 h by the participants, out of phase of their usual sleep and mealtimes. The resulting misalignment caused a reduction in leptin (−17%), an increase in blood glucose (+6%) in spite of increased insulin (+22%), an absolute reversal of the circadian pattern of cortisol, increased mean arterial blood pressure (+3%), and a decrease in sleep efficiency (−20%).

A subsequent experimental study under controlled conditions examined the effects of circadian disruption (28-h day forced desynchrony protocol) accompanied by extended sleep curtailment (5.6 h/24 h), on blood glucose levels, in 21 participants, for 3 weeks (14). The investigators reported a reduction in resting metabolic rate and a rise in plasma glucose concentrations following a meal; these changes were secondary to inadequate pancreatic insulin secretion.

However, does circadian rhythm disruption affect the cardiometabolic system if sleep duration is maintained? Leproult et al. tried to answer this question by studying 26 healthy volunteers using a parallel group design. The experimental protocol comprised 3 days with a sleep duration of 10-h, followed by 8 days of sleep curtailment to 5 h with pre-set bedtimes at night (circadian alignment), or with a delay in bedtimes by 8.5 h on 4 of the 8 days (circadian misalignment) (15). Insulin sensitivity was reduced in both groups (the circadian aligned and misaligned groups) without a compensatory rise in insulin secretion (15). Moreover, C-reactive protein (CRP), which is a sensitive marker of inflammation that predicts increased risk of coronary heart disease, increased with circadian misalignment, independent of sleep loss.

The above laboratory-controlled studies suggest that acute misalignment of the circadian rhythm increases the risk of developing cardiometabolic disorders. Nevertheless, studies in the general public are required to supplement these short-term laboratory-based results of forced circadian desynchrony. Moreover, long-term studies are needed to explore the possibility of adaptation to the changes of chronic circadian misalignment (16).

## Mealtime and Cardiometabolic Risk

Increasing evidence indicates that mealtime has a significant role in metabolic regulation and that mealtime closely interacts with the circadian clock (17). “Chrononutrition,” which means meal timings, is a newly proposed discipline that addresses the interaction between mealtime, circadian clock, and metabolism.

Current evidence suggests that mealtime affects circadian rhythm, metabolism, and body weight (18). Eating during the wrong time of the day results in a misalignment between the peripheral circadian clocks and the central biologic clock in the SCN. The resulting desynchronization increases the risk of developing cardiometabolic diseases (7, 17). Nocturnal species eat most of their daily food requirement at night. For example, mice consume most of their daily intake of food (70–80%) during the dark phase (active phase) (19). Therefore, when mealtime is confined to the light phase (inactive phase) of the day, uncoupling between peripheral and central clocks occurs, and mice gain more weight compared to their counterparts fed during the dark phase in as little as 1 week (20). Additionally, another study in mice demonstrated that restricting food availability to the active phase (8–9 h) had a protective effect against weight gain and metabolic syndrome, secondary to atherogenic food consumption (21). Although, the protective effect of restricting food to the active phase is not only secondary to caloric restriction. Hatori et al. exposed mice to either *ad libitum* or restricted their feeding time to 8 h per day (active phase) of high-fat-diet (22). Interestingly, mice with restricted feeding time consumed comparable calories to their counterparts with *ad libitum* food access, yet food restriction to 8 h had a protective effect against weight gain, increased insulin levels, hepatic steatosis, and systemic inflammation (22).

Similarly, in humans, studies have shown very similar outcomes, where eating at the wrong time (nighttime “inactive phase”) is associated with a higher risk of developing cardiometabolic dysfunction (7). In a Swedish study of 3,610 men and women, eating late at night was linked to an increased obesity odd ratio (OR) of 1.62 (95% confidence interval [CI], 1.10–2.39) compared to those who did not eat late at night (23). A recent systematic review and meta-analysis of 10 observational and experimental studies that assessed the effect of meal timing on obesity and metabolic alterations in humans reported negative impact of late meal timing on weight and metabolism (24). Additionally, both observational and experimental studies reported an association of late mealtime with hyperglycemia and diabetes mellitus (24).

In shift workers, studies have revealed an association between night eating and metabolic disturbances. In a study of 11 female nurses over 3 days of nightshift duties, standard meals of 440 kcal were served at 07:30, 23:30, and 03:30; and the glycemic response was then assessed over 4 h (25). Although baseline glucose levels were similar before each meal, postprandial levels were highest after meal intake at 23:30. Moreover, the highest insulin level was reported after meal intake at 23:30, and the lowest after meal intake was at 03:30. In another earlier randomized study, with a cross-over design, eight non-obese males were subjected to day or night work for a single shift with fixed caloric intake and fixed proportions of proteins, fats, and carbohydrates (26). Meals were served according to the day and night protocols at 01:00/13:00 and 07:00/19:00, and the snacks at 04:00/6:00. Blood samples for measurements were collected after an overnight fast, and at 8 h following the first meal in each protocol. The study demonstrated increased postprandial triacylglycerol and glucose levels in the night protocol (26).

To investigate the effect of restricted food intake to daytime or nighttime on cardiometabolic risk, non-obese (*n* = 27) participants were asked to abstain from food between 19:00 and 06:00 for 2 weeks or to continue their usual eating routine (27). The study showed that total caloric intake when eating was restricted to daytime was associated with a 0.4-kg weight loss compared with a 0.6-kg weight gain during the control period. A recent study tested the effect of early TRF as a form of TRF, which entails eating early in the day to align with human circadian rhythms in metabolism in eight men with prediabetes (28). In a randomized crossover design, the participants were assigned to early TRF (6-h period for eating, with dinner before 15:00) or a control group (12-h period for eating); and the participants were followed-up for 5 weeks, following which, they did a crossover to the other schedule. The study demonstrated that early TRF (6-h feeding period, with dinner before 3 p.m.) led to reduced blood pressure, oxidative stress, and appetite, and increased insulin sensitivity and β cell responsiveness compared to the controls (12-h feeding period) for 5 weeks (28). Another two recent studies assessed the effect of early TRF (08:00–14:00) vs. a control protocol (08:00–20:00) in a randomized crossover design on 11 overweight adults (28, 29). Early TRF significantly reduced the mean 24-h glucose levels and glycemic excursions (29). Moreover, the early time-feeding protocol led to decreased appetite without affecting energy expenditure (30).

### Breakfast and Cardiometabolic Risk

A study compared the effects of late dinner served at 23:00 with dinner served at 18:00 on 12 female university students (31). Blood glucose level was significantly higher after breakfast under the late dinnertime protocol. On the other hand, Jakubowicz et al. assessed the effect of a high-energy breakfast in non-obese women with polycystic ovary syndrome over a period of 90 days (32). The participants were randomized into two isocaloric (1,800 kcal/day) diets divided over three meals with different caloric distribution: (1) a breakfast diet group, which served 980 kcal at breakfast, 640 kcal at lunch, and 190 kcal at dinner; or (2) a dinner diet group that provided 190 kcal at breakfast, 640 at kcal lunch, and 980 kcal at dinner. The study revealed that the group with a high caloric intake at breakfast and lower calories at dinner had an improvement in insulin sensitivity parameters and a reduction in cytochrome P450c17α activity (32).

However, the beneficial effect of a breakfast meal on weight and cardiometabolic health is not unanimously reported in all published work. A recent meta-analysis included 12 randomized controlled trials from rich countries, and aimed to assess the impact of regular breakfast ingestion on weight and energy consumption in adults (33). The paper reported a small difference in weight favoring participants who were not assigned to breakfast (mean difference 0.44 kg, 95% CI: 0.07–0.82; mean follow-up 7 weeks; range 2–16 weeks). However, there was inconsistency across trial results, and participants in the breakfast group had a higher total daily energy consumption (~260 extra calories/day) compared to those assigned not to take breakfast (mean difference 259.79 kcal/day). Additionally, the quality of the analyzed studies was generally low; therefore, the authors concluded that the results of their study should be interpreted with caution (33). Moreover, Gonzalez et al. conducted four trials in a randomized cross-over design on 12 healthy men where they performed overnight fasting followed next day by one of the following protocols: (1) rest without breakfast; (2) exercise without breakfast; (3) breakfast followed by rest; or (4) breakfast followed by exercise (34). The study demonstrated that after 1 day of monitoring, energy intake from breakfast and energy expenditure from exercise were not compensated for at lunch. Accordingly, energy balance was most positive following breakfast and rest and least positive following breakfast omission and exercise. Nevertheless, the findings of this study should be interpreted with caution as it does not predict the longer-term outcomes of energy and fat balance due to its single-meal design (34).

Additionally, data from studies that assessed the effects of Ramadan diurnal IF (see below) on weight and cardiometabolic risk, where individuals take breakfast 30 min before dawn and dinner after sunset, demonstrated that this diurnal IF protocol reduces weight and cardiometabolic risk (4, 35).

This inconsistency indicates that large randomized controlled trials of high quality are needed to assess the effect of breakfast meal on weight and cardiometabolic risk. A recent consensus statement from the American Heart Association concluded that epidemiological data suggest a probable harmful effect of late mealtime on cardiometabolic risk. However, clinical interventional investigations that could address causality have been limited and not too focused to allow definite conclusions to be drawn, in order to formulate recommendations (7). Additionally, the consensus statement indicated that mealtime and frequency are not the only culprits; the duration between meals and the amount of caloric intake consumed in each meal are as important (7).

## Intermittent Fasting and Cardiometabolic Risk

IF means abstinence of food and drink in specified time periods. IF can be practiced in different forms, such as abstinence from food every other day (36), significant reduction in caloric consumption every other day (37), moderate caloric restriction for 2 consecutive days/week (38), restricting of food to specific times of a 24-r period, which is called TRF (21), abstinence from food for 1 or 2 days per week and then *ad libitum* food consumption for the rest of the week, as well as the diurnal IF performed by Muslims during the month of Ramadan where fasting performers abstain from food as well as drinks from dawn to sunset for the whole month (29–30 days) (39).

Researchers have developed a great interest in the health effects of IF over the past decade. Nevertheless, there is still a scarcity of long-term studies and studies that control for confounders that may affect the measured health indices, while well-designed studies included small numbers of participants.

Current evidence suggests that experimental IF is associated with weight loss, although there are no data on the long-term effects of this practice on weight. Moreover, recent data demonstrated that experimental IF lowers blood pressure (4). Data from animal studies suggest that experimental IF may have cardioprotective effects (4). Nevertheless, most of the studies that assessed the effect of experimental IF on cardiometabolic risk have major limitations. The sample sizes of most previous studies were relatively small. Additionally, most of the studies that assessed the effects of experimental IF on cardiometabolic risk did not control for major confounders that may have an impact on the measured markers, such as sleep duration and timing, energy expenditure, and light exposure (40). Furthermore, some of the proposed experimental fasting protocols, such as the alternate-day fasting protocol are not practical due to accompanying severe hunger (41). As there are major differences between Ramadan diurnal IF and other forms of experimental IF, in this article, we restricted the discussion to Ramadan diurnal IF (42).

### Ramadan Diurnal Intermittent Fasting

Diurnal IF during Ramadan month is the fourth pillar of Islam and has been practiced by all Muslims for over 1,400 years. Ramadan is the ninth month of Islamic (*Hijri*) year, which follows the lunar system. The lunar year is 11 days shorter than the Gregorian year; therefore, Ramadan occurs in a different season every 9 years. The season during which Ramadan occurs affects the duration of each fast, because fasting hours are longer in summer than in winter. This, in turn, may affect sleep patterns, due to factors, such as shorter nights and earlier dawns. Besides, the climate may affect sleep. During the hot summer, many people resort to napping in the middle of the day, which may influence night sleep. Moreover, the geographical location may affect the duration of fasting. As one moves further from the equator, daytime becomes longer in summer and shorter in winter (43). These factors may influence sleep and circadian rhythm. Therefore, when studying sleep patterns during Ramadan, it is essential to document the time of year, the location of the study, and the times of dawn and sunset.

The abstinence of food and drink during the daytime results in sudden shift in meal timing to the period between sunset and dawn.

**Figure 1:**
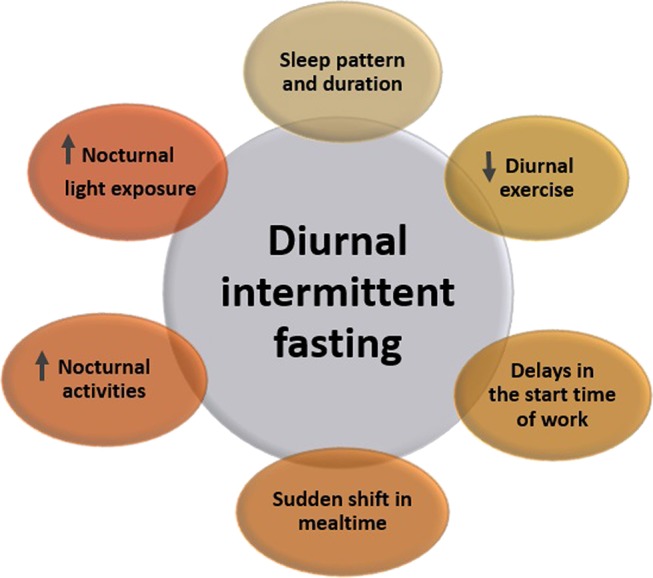
Lifestyle changes that accompany the month of Ramadan and may influence the cardiometabolic risk.

Theoretically, restricting feeding to a limited time of the 24-h day is expected to result in reduced energy intake, and this is the essence of diurnal IF during Ramadan (42). Ideally, in Ramadan, fast performers are expected to eat two main meals, breakfast at sunset and a pre-dawn (suhur) meal ~30 min before dawn, and to get a good night sleep. This practice is expected to result in weight loss and improved physiological parameters that are related to increased weight. However, in real life, this does not always occur as many lifestyle and cultural factors interact with the IF practice during Ramadan (Figure 1) (4, 42). In other words, Ramadan fasting can be considered as a TRF schedule without intentional restrictions on energy consumption, where two large meals are administered; one at sunset and the other in the early morning before dawn, and adequate sleep at night (44).

Therefore, the sleep and feeding behavior of Ramadan is different from a night shift schedule, which has been shown to be associated with cardiometabolic disorders (11, 12). Religiously, this practice aims to persuade fast-performers to get up early for the pre-dawn meal, which is followed by the dawn prayer (4).

Another important point that should be addressed when discussing the impact of Ramadan intermittent fasting on cardiometabolic risk is physical activity during Ramadan. A recent meta-analysis of studies that did not attempt to influence physical activity revealed that measures of physical activity did not indicate any significant change between pre-Ramadan and during Ramadan of either maximum effort physical activity or daily physical activity (35).

Ramadan fasting has unique characteristics, which include the long duration of the practice for 1 month. This long duration may cause some adaptations to the new behavior. Therefore, physiological changes in the first week of the month may not be similar to changes at the end of the month. Additionally, the geographic location away from the equator affects the duration of light and dark cycles and hence the fasting hours.

### Ramadan Diurnal Intermittent Fasting and Cardiometabolic Changes

#### Effects on Circadian Rhythm

During Ramadan fasting, there is a sudden shift in mealtime from mainly daytime to the dark hours between sunset and dawn. Therefore, it is logical to ask whether this sudden shift in mealtime will affect the circadian pattern of the body, as this may have impact on the cardiometabolic system.

Reviewing all studies that aimed to assess the circadian rhythm changes during Ramadan reveals two types of studies: (1) studies that were conducted in a non-constrained environment and did not control for lifestyle changes, caloric intake, and nocturnal meal timings, and these studies reported rapid and significant delays in bedtime and rising time (45–47); and (2) recent studies that accounted for sleep/wake schedule, sleep duration, light exposure, caloric consumption, meal timings and energy expenditure, which demonstrated no changes in circadian rhythm during fasting (48–50). In the second group, meals were served early at night, the participants slept early according to their usual routine and then woke up for the predawn meal in the early morning.

In an earlier study, our group examined the changes in circadian pattern of the proximal skin temperature (an indicator of core body temperature) before Ramadan and during the first 2 weeks of Ramadan in an unconstrained environment using a validated portable device in six young adults who had an evening chronotype (i.e., the participants slept during daytime and wake up to eat at night before Ramadan) (47). Despite no changes in the meal timings during Ramadan, the acrophase of the proximal skin temperature was delayed; indicating a shift-delay in the circadian clock (47). Another study in Saudi Arabia in the free-living environment reported flattening of the cortisol rhythm, which is known to be linked to chronic stress or endogenous hypercortisolism and associated with insulin resistance, which supports that lifestyle changes that accompany Ramadan month are associated with disruption of the circadian rhythm (51).

These findings indicate that the shift delay in the biologic clock reported in some studies during Ramadan is not only due to mealtime shift, but other factors. Lifestyle changes may influence the biologic clock during Ramadan. Therefore, based on the available evidence, restricting mealtime to the early evening and predawn times, and getting a good night sleep during Ramadan does not affect the biologic clock.

#### Effects on Weight and Lipid Profile

A meta-analysis that assessed the weight changes in fast performers during and after Ramadan diurnal IF including 25 studies (mainly from West Asia) in a free-living unconstrained environment reported significant weight loss at the end of the month (−1.24 kg; CI: −1.60, −0.88 kg). However, weight was regained 2 weeks after Ramadan (52). Another meta-analysis that included 30 cohort studies of healthy young men and women in an unconstrained environment assessed the impact of Ramadan diurnal IF on weight, lipid profile, and blood glucose level (53). The analysis revealed a reduction in low-density lipoprotein (−1.67; CI: −2.48 to −0.86) and fasting blood glucose levels (−1.10; CI: −1.62 to −0.58) in both genders (53). The meta-analysis of 21 studies that assessed the effects of Ramadan fasting on body weight, included 830 participants of both genders (men = 531 women = 299) (53). There was a slight but significant reduction in weight in men (−0.24 [−0.36, −0.12], *p* = 0.001), but no significant changes were documented in women (−0.04 [−0.20, 0.12], *p* = 0.620) (53).

A more recent meta-analysis assessed the effect of Ramadan IF on weight and body composition (35). The analysis included 70 studies (~3,000 participants) and demonstrated a significant positive correlation between pre-Ramadan body mass index (BMI) and weight lost during Ramadan. Fasting resulted in a significant reduction in weight (−1.34 [95% CI: −1.61 to −1.07] kg, *p* < 0.001), despite not giving the participant any advice on lifestyle or dietary modifications (35). Additionally, there was a significant decrease in the percentage of fat in overweight and obese participants (−1.46 [95% confidence interval: −2.57 to −0.35] %, *p* = 0.010) (35). This reduction was not seen in normal-weight subjects (35). Nevertheless, weight and body composition returned to about the pre-Ramadan levels after 2–5 weeks of follow-up.

Another recent meta-analysis included 33 studies and assessed the effect of Ramadan diurnal IF on lipid and lipoprotein in ~2,000 healthy participants, with no lifestyle modifications (54). The weighted mean changes at the end of Ramadan demonstrated a significant reduction in low density cholesterol levels in men (weighted mean difference [WMD] = 2.65 mg/dl; 95% CI = 5.16, −0.14) but not in women (WMD = 9.50 mg/dl; 95% CI=21.93, 2.92) (54).

One more study assessed the effect of Ramadan IF in a free unconstrained environment on 10 obese men (7 of them had metabolic syndrome) (55). The study reported that by the end of the month of Ramadan, BMI had decreased significantly and was associated with a significant reduction in homeostasis model assessment of insulin resistance (HOMA-IR) and fasting levels of blood glucose (55).

A recent systematic review and meta-analysis assessed the impact of Ramadan IF on metabolic syndrome components in healthy, non-athletes and included 85 studies (~4,500 participants) that were performed in 23 countries (56). The study reported small but significant reductions in waist circumference, fasting blood glucose level, systolic blood pressure, and triglyceride level, and a small increase in HDL cholesterol (56).

Nonetheless, it is imperative to mention here that all the above reviewed studies occurred in the free-living unconstrained environment and did not account for potential confounders that may have affected the measured parameters, such as caloric intake and food composition (54), sleep duration (55), physical activity (57), and energy expenditure (57), which have been shown to change in Ramadan (39).

#### Effects on Diabetes

A study that assessed changes in fat mass and metabolic markers in 29 patients with type 2 diabetes for 15–21 days during Ramadan reported a significant reduction in glycated hemoglobin (HbA1c) (8.6 ± 2.4% at baseline vs. 8.0 ± 2.3% toward the end-Ramadan, *p* = 0.02) (58). Moreover, there was a significant decrease in body fat mass, but no changes were detected in body weight (58). This study suggests that Ramadan diurnal IF may improve glycemic control in patients with diabetes. Nevertheless, it is important to indicate here that patients with diabetes must consult their physicians before fasting.

A new study assessed changes in 228 patients with type 2 diabetes who were on >3 medications for diabetes before and after Ramadan (59). The study reported a significant decrease in HBA1c [7.8% (62 mmol/mol) vs. 7.6% (60 mmol/mol); *p* = 0.004] after Ramadan (59).

#### Effects on Blood Pressure and Cardiovascular System

Al-Shafei examined the effects of Ramadan IF on arterial pulse pressure (PP) in 40 patients with hypertension, without receiving any instructions on behavioral or dietary modifications (60). The results revealed a significant decrease in PP (17.2%), with no changes observed in the control group (60).

A recent study assessed blood pressure changes during Ramadan diurnal IF on 60 subjects with prehypertension or hypertension but who did not use antihypertensive agents (61). The study revealed that during Ramadan, there was a significant reduction in blood pressure, including the 24-h ambulatory blood pressure monitoring (61).

A recent systematic review and meta-analysis of studies that assessed the incidence of cardiovascular events during Ramadan that included 15 studies reported no significant increase in the incidence rate of acute cardiac conditions, including congestive heart failure, myocardial infarction, and stroke during Ramadan (62).

#### Effects on Inflammatory Biomarkers

There is a strong association between inflammatory biomarkers and increased risk of metabolic and cardiovascular disorders (63–65). In the past few years, researchers interest was increased toward studying the effects of Ramadan diurnal IF on inflammatory biomarkers.

A recent systematic review and meta-analysis included 12 studies (from 8 countries) and assessed changes in inflammatory biomarkers in healthy subjects pre and post-Ramadan (66). The pooled data revealed that diurnal IF during Ramadan was associated with a very small decrease in interleukin-1 (IL-1) (Hedge's *g* = 0.016); CRP (Hedge's *g* = 0.119); and a small reduction in tumor necrosis factor-alpha (TNF-α) (Hedge's *g* = 0.371); and IL-6 (Hedge's *g* = 0.407) (66).

Shariatpanahi et al. conducted a study on 65 patients with metabolic syndrome (who were admitted to a hospital), to evaluate the effect of diurnal IF during Ramadan on CRP and fibrinogen levels in the unconstrained environment. Participants were served two meals, at sunset and 30 min before dawn (fasting period 17 h) (67). At the end of the month, there was a significant reduction in fibrinogen, CRP, BMI, and waist circumference compared to the baseline (67). The findings of this study suggest that limiting the mealtime during Ramadan to sunset time and early morning (pre-dawn) and obtaining good nocturnal sleep may help in maintaining good alignment between the central and peripheral biologic clocks and have beneficial physiological effects. Similar reductions in IL-1β, IL-6, and TNF-α have been reported in young, healthy volunteers (68).

An additional study assessed inflammatory biomarkers in 50 healthy adults (21 men and 29 women) and reported a significant decrease in the circulating levels of interleukin (IL) IL-1β, IL-6, and TNF-α occurred toward the end of the third week of Ramadan (68). Another recent study assessed specific cardiometabolic risk factors twice (9 a.m. and 9 p.m.) before Ramadan and the end of the second week of Ramadan in 23 volunteers and reported improvements in serum high sensitivity CRP, gamma glutamyl transferase, and IL-1 (69).

In a recent study, Faris et al. assessed the influence of IF during Ramadan on visceral adiposity (using three-dimensional magnetic resonance imaging [3D-MRI]) and inflammatory biomarkers in 61 obese subjects (70). At the end of Ramadan, visceral fat tissue area, weight, and systolic blood pressure were significantly decreased compared to the baseline (70). Visceral adiposity has been shown to have a significant association with cardiometabolic risk, even in non-obese individuals (71, 72). Moreover, Faris et al. reported that serum levels of adiponectin, IL-6, TNF-a, and insulin-like growth factor-1 were significantly decreased. However, there was a significant increase in the serum levels of visfatin, leptin, apelin, and IL-10, at the end of Ramadan (71, 72). Previous studies have reported contradictory results regarding the effect of diurnal IF on leptin levels. While an earlier study reported an increase in serum leptin by 133% (73); another study reported no changes in leptin levels during Ramadan IF (74). However, a major limitation of all previous studies is that only a single blood sample for leptin was taken. It is known that leptin levels follow a circadian rhythm and are affected by meals (75); therefore, the levels of measured serum leptin will depend on the timing of the sample collection in relation to the timing of the last meal. Therefore, our group assessed serum leptin levels at 22:00, 02:00, 04:00, 06:00, and 11:00 at baseline; while performing diurnal IF and reported a significant decrease in leptin levels at 22:00 and 02:00 compared with the baseline concentrations (at 22:00: 194.2 ± 177.2 vs. 146.7 ± 174.5; at 02:00: 203.8 ± 189.5 vs. 168.1 ± 178.1; *p* < 0.05) (76).

Based on the above, most studies have demonstrated reductions in inflammatory biomarkers during diurnal IF. However, the above papers have major shortcomings that need to be kept in mind. A single morning sample for the measured biomarkers does not take the circadian variation of the levels of the measured biomarkers into consideration (77). Moreover, the above studies were conducted in the free-living environment, which means that there was no control to determine the influence of potential confounders related to lifestyle changes during Ramadan month that may affect the measured biomarkers, such as energy consumption (78), sleep duration (79), and physical activity (80).

To avoid the above limitations, our group conducted an experiment on 12 young, healthy males to assess the inflammatory biomarkers before and during Ramadan diurnal IF while controlling for the above-mentioned confounders (81). We measured the levels of cytokines (IL-1β, IL-6, and IL-8) at 22:00, 02:00, 04:00, 06:00, and 11:00 and demonstrated a significant reduction in cytokines levels across the 24 h period during diurnal IF compared to the baseline, mainly with IL-1β and IL-6.

#### Effects on Oxidative Stress

A few studies assessed the effects of Ramadan diurnal IF on oxidative stress, which revealed contradictory findings. Some investigators demonstrated a significant decrease in lipid peroxidation (60, 82); while others reported no changes (83–85).

The pooled data of malondialdehyde (MDA), in a recent systematic review and meta-analysis that included four studies, which assessed the effects of Ramadan diurnal IF on oxidative stress, reported a very small decrease in MDA Hedge's g (*N* = 117, *K* = 4) of 0.219 (*I*^2^ = 0.0%). However, the sensitivity analysis for MDA demonstrated that the results were influenced by one study (86), and Hedge's g increased to 0.4 when the investigators removed that study.

In a previous study on 50 healthy volunteers, Faris et al. demonstrated that during Ramadan diurnal IF, markers of oxidative stress were significantly decreased in those who experienced weight loss (85). Therefore, it is difficult to figure out if the reduction in oxidative stress was due to weight loss or IF. In a recently published study, Madkour et al. examined the effects of Ramadan diurnal IF on the expression of cellular metabolism genes (Sirtuin [SIRT] 1 and SIRT3) and antioxidant genes (Mitochondrial transcription factor A [TFAM], superoxide dismutase-2 mitochondrial [SOD2], and nuclear respiratory factor-2 [Nrf2]) in 56 healthy overweight and obese subjects and 6 normal BMI controls (87). The sirtuins are a family of nicotine adenine dinucleotide (NAD)^+^-dependent deacetylases, which work as cellular sensors to discover energy availability and modify metabolic processes, and have been reported to control several genes (88). Expression of these genes functions as endogenous reactive oxygen species scavengers, and hence protect the cells from oxidative stress damage (88). Madkour et al. reported that at the end of Ramadan, the expression of the antioxidant genes (TFAM, SOD2, and Nrf2) was significantly increased in obese subjects compared to the control group (87). On the other hand, there was a significant decrease in the SIRT3 gene (metabolism-controlling gene) toward the end of Ramadan, with a non-significant reduction in SIRT1 gene (87). The investigators concluded that Ramadan diurnal IF probably provide a protective antioxidant effect in obese patients. The investigators also proposed that the reduction in SIRT1 and SIRT3 could be explained by the fact that the sirtuins levels usually increase secondary to inflammation; the reduction in the inflammatory activity during Ramadan diurnal IF may result in a decrease in sirtuins levels (87).

As with the inflammatory biomarkers, measured biomarkers of oxidative stress are affected by the above discussed lifestyle changes that accompany Ramadan (89–96), and the relationship with the timing of the blood samples to mealtime (90). Previously discussed studies collected only a single early morning blood sample. Oxidant levels could have been affected by the sample collection time as well as by the interaction between sample collection time and mealtime (91, 93). The blood sample collection time of oxidant or anti-oxidant activity may affect the measured levels. Blood samples collected in the early morning after an overnight fast (at baseline, before Ramadan) may be different from blood samples collected after a pre-dawn (Suhur) meal during Ramadan (97). During fasting, there is a larger utilization fat instead of glucose as an energy source (85), which may cause higher fat oxidation.

To avoid the above limitations, our group examined the impact of Ramadan diurnal IF during and outside of Ramadan on the circadian changes in MDA while accounting for the above discussed confounders (97). MDA levels were measured at 22:00, 02:00, 04:00, 06:00, and 11:00, while controlling for meal composition and caloric ingestion, light exposure, physical activity, sleep duration, and body weight monitoring (no change in weight) during the study period in a sample of healthy young males (97). The resulted demonstrated that Ramadan diurnal IF does not alter serum MDA levels in healthy subjects.

Based on the above, no definitive conclusion about the impact of Ramadan IF on oxidative stress can be formulated. Large scale studies are needed to assess oxidative stress during diurnal IF while controlling for discussed confounders.

## Summary

Recent data suggest that IF, circadian rhythm, and meal timings may influence cardiometabolic physiology and risk of cardiometabolic disorders. TRF and mealtime are known to affect circadian rhythm and synchronization, between the central and peripheral biologic clocks.

Desynchronization of the circadian system has been linked to several cardiometabolic disorders, including impaired glucose tolerance, reduced sensitivity to insulin, increased markers of systemic inflammation, increased blood pressure, decreased energy expenditure, and increased weight.

The discipline “chrononutrition” has been recently proposed to address the interaction between mealtime, circadian clock, and metabolism. Animal studies indicate that eating during the wrong time of the day (inactive phase) causes a misalignment between the peripheral circadian clocks and the central clock in the SCN, which increases the risk of developing cardiometabolic diseases. Likewise, several human studies indicated an association between late meal timing and a greater risk of poor cardiometabolic health. Nevertheless, clinical interventional studies that could address causality between late mealtime and cardiometabolic morbidity have been limited and not too focused to allow definitive conclusions to be drawn.

Currently available evidence indicates that Ramadan diurnal IF does not affect the circadian rhythm when meal timings are confined to the early evening and predawn periods, with an adequate night sleep. Moreover, Ramadan diurnal IF has beneficial effects on weight, lipid profile, glycemic control, inflammatory biomarkers, and probably oxidative stress. Nonetheless, Ramadan diurnal IF is accompanied by several lifestyle changes that may affect all the markers of cardiometabolic diseases. Therefore, larger studies that account for various confounding variables are needed to examine the influence of Ramadan diurnal IF on markers of cardiometabolic disorders.

## Author Contributions

AB and AA have contributed equally to review of the literature, analysis, and writing of the manuscript.

### Conflict of Interest

The authors declare that the research was conducted in the absence of any commercial or financial relationships that could be construed as a potential conflict of interest.

## Abbreviations

3D-MRI: three-dimensional magnetic resonance imaging
BMI: body mass index
CI: confidence interval
CRP: C-reactive protein
HBA1c: glycated hemoglobin
HOMA-IR: homeostasis model assessment of insulin resistance
IF: intermittent fasting
IL: interleukin
MDA: malondialdehyde
NAD: nicotine adenine dinucleotide
Nrf2: nuclear respiratory factor-2
OR: odd ratio
PP: pulse pressure
SCN: suprachiasmatic nucleus
SIRT: sirtuins
SOD2: superoxide dismutase-2 mitochondrial
TFAM: Mitochondrial transcription factor A
TNF-α: tumor necrosis factor-alpha
TRF: time-restricted feeding
WMD: weighted mean difference.
